# A Practical Suggestion for the Prevention of Ankylostomiasis

**Published:** 1905-01

**Authors:** 


### A PRACTICAL SUGGESTION FOR THE PREVENTION OF ANKYLOSTOMIASIS.

Of the many troubles with which the planter in tropical countries has to contend, not the least is coolie anemia, and the disabling form of pustular dermatitis variously called coolie itch, ground itch, etc.

The dependence of coolie anemia on ankylostoma infection has long been recognized, and the probability that the form of dermatitis called coolie itch is produced by the penetration of the skin of the feet and legs by the embryos of ankylostoma contained in contaminated soil is now, from the observations and experiments of Looss and Bentley, practically a certainty. We are therefore in a position to indicate with precision the direction of prophylactic measures against both phases of this infection. Unfortunately, although it is easy enough to indicate measures which theoretically would be efficient, to get them applied is a very different matter. At the suggestion of Bentley, boots and baths of carbolic lotion have been tried, but so far apparently without striking results, in great measure in consequence of the want of co-operation on the part of the coolies.

Some time ago, in the course of a conversation with a West Indian sugar-planter, I learned of a method which in his hands had proved efficient; it appears to me to meet the circumstances, and deserves, I think, publicity and further trial. This gentleman, in ignorance of Looss's and Bentley's observations, had concluded that coolie itch was contracted from contact with fecally-fouled soil, and that somehow it led up to coolie anemia—that is, ankylostoma anemia; he reasoned that, could he prevent the injurious effects of the contaminated soil, he would not only prevent coolie itch, but the consequent anemia. Knowing the antiseptic and adhesive properties of green Barbadoes tar (a mineral oil rich in paraffin, and cheap, costing 3d. a gallon), he got his overseers to see that the coolies, before going out to work in the contaminated fields, dipped their feet and legs in a bucket of this tar, and then walked across and through some fine sand; in this way an impervious sandal and stocking was donned every morning. In practice it proved efficient. My informant suggests that sawdust would probably be better than sand. He told me he got the idea from a practice existing among goose farmers in certain parts of Germany, who, by first dipping the feet of their geese in tar and then in fine sand, provided the birds with a plastic and protective sandal, thereby enabling them to be driven many miles to market without injury.—*Patrick Manson in British Medical Journal.*

---

JUST at this season of the year we are especially called upon to consider the advantages to be found in Glyco-Thymoline for the treatment of acute catarrhal diseases of the nose and throat.

Coryza, nasopharyngitis, tonsillitis and laryngitis are now most common. After exposure to cold or damp chill the mucous membrane, with its delicate cell-structure and fine capillary network, takes on a turgid appearance. The minute blood-vessels or capillaries become congested and their function practically suspended. The blood-cells through lack of nourishment die and are thrown off. The glandular secretions are altered; instead of excreting a bland, non-irritating mucus, we have present an acid discharge most irritating in type. This is about the condition we find in all catarrhal inflammations.

How does Glyco-Thymoline apply here? What are its special advantages?

When applied warm in a twenty-five per cent. solution, Glyco-Thymoline gives a soothing sensation to the inflamed membrane, due to its anesthetic or anodyne properties.

Glyco-Thymoline quickly dissolves all accumulations of thick, ropy mucus, crusts, formations, etc.

Glyco-Thymoline in a twenty-five per cent. solution, being approximately of the same alkalinity and specific gravity as blood-serum, causes by its exosmotic action (the passage outwardly through the tissues of normal secretions and products of inflammation), a rapid depletion of the engorged tissue, thus aiding nature after her own manner in restoring capillary circulation, normal glandular action, and fostering cell nourishment, which soon brings about a general normal condition to the membrane.

